# Occult Perforation of the Esophagus during Removal of an Enteral Feeding Tube: A Case Report and Literature Review

**DOI:** 10.1155/2023/4230158

**Published:** 2023-03-29

**Authors:** Mohammad Alabdallat, Gustav Strandvik, Ibrahim Afifi, Ruben Peralta, Ashok Parchani, Ayman El-Menyar, Sandro Rizoli, Hassan Al-Thani

**Affiliations:** ^1^Department of Surgery, Trauma Surgery, Hamad Medical Corporation, Doha, Qatar; ^2^Department of Surgery, Universidad Nacional Pedro Henriquez Urena, Santo Domingo, Dominican Republic; ^3^Department of Clinical Medicine, Weill Cornell Medical College, Doha, Qatar

## Abstract

*Background*. The use of oral or nasal route for enteral feeding is a standard practice in intensive care patients with a safe profile in general. However, complications associated with the insertion of a nasogastric (NGT) or orogastric tube (OGT) are common in the medical literature compared to the removal of such tubes. *Case presentation*. We presented a 38-year-old male who was involved in a motor-vehicle collision and found with low Glasgow Coma Scale outside his vehicle. He had polytrauma and was intubated—and commenced on enteral feeding via an OGT. Esophageal bezoar developed within a few days around the feeding tube, resulting in significant force being required to remove it, which was complicated by esophageal perforation. The esophageal injury was treated conservatively with uneventful recovery. *Discussion and conclusions*. Although limited case reports of esophageal enteral feeding bezoar formation do exist in the literature, we believe that this is the first case report of esophageal perforation due to the forceful removal of a wedged OGT secondary to esophageal bezoar formation. Morbidity associated with OGT/NGT is not common and may require a high index of suspicion to be identified. This is especially true if resistance is appreciated while removing the NGT/OGT. Gastroenterology consultation is recommended as early as possible to detect and manage any complications, however, their role was very limited in such stable case. In addition, early computed tomography (CT) can be considered for timely recognition of esophageal perforation. Non-operative management may be considered in stable patients, especially if the leak is in the cervical portion of the esophagus. Finally, prevention is better than cure, so being diligent in confirming NGT/OGT position, both radiologically and by measuring the tube length at the nostril/mouth, is the key to avoid misplacement and complication. This case raises the awareness of physician for such preventable iatrogenic event.

## 1. Background

Bezoars are mass lesions of the gastrointestinal tract, which are composed of substances such as undigested food, medication, and hair [1]. Four basic types of bezoars have been defined: *phytobezoar*, *trichobezoar*, *pharmacobezoar*, and *lactobezoar* [2]*. Bezoars* have clinical importance because they may be associated with complications such as bleeding, intestinal obstruction, perforation, and fistula formation to adjacent organs or skin. Bezoars can be found anywhere in the gastrointestinal tract, although the incidence in the esophagus is rare. Esophageal bezoars are classified as either primary or secondary, and generally present with dysphagia, retrosternal pain, or gastroesophageal reflux [3].

A search of the literature in English language (PUBMED) using the words (esophageal bezoars, perforation) revealed 84 related articles, 42 reported cases of feeding formula-associated esophageal bezoars and no reported cases of esophageal perforation occurred during removal of enteral feeding tube that stuck to an esophageal bezoar. The formation of feeding formula bezoars is triggered by acidic gastroesophageal reflux. The acidic pH in the esophagus in turn causes clotting of the casein in the formula [4]. Predisposing factors for bezoar formation include mechanical ventilation, supine position, neurological diseases, diabetes mellitus, hypothyroidism, obesity, and history of partial gastrectomy [4–7]. We present a rare case of esophageal perforation due to forceful removal of a sticky orogastric tube (OGT), caused by an upper esophageal bezoar. The bezoar likely developed due to an unnoticed proximally situated OGT opening at the level of the gastroesophageal junction; a casein-containing feed was administered.

## 2. Case Presentation

A 38-year-old male was involved in a motor-vehicle collision and found with a Glasgow Coma Scale (GCS) of 3/15 outside his vehicle. He was intubated by the emergency medical services (EMS) at the scene before being transferred to the level 1 trauma center. On arrival to the emergency department (ED), the patient was hemodynamically unstable with a left-sided tension pneumothorax and a positive Focused Assessment with Sonography for Trauma (FAST) test. An emergency thoracostomy tube (TT) was inserted, and the patient was taken to the operating room (OR) for emergent laparotomy. Splenectomy was performed for ruptured spleen. The abdomen was closed, and the patient underwent pan-computed tomography (CT) scan.

**Figure 1:**
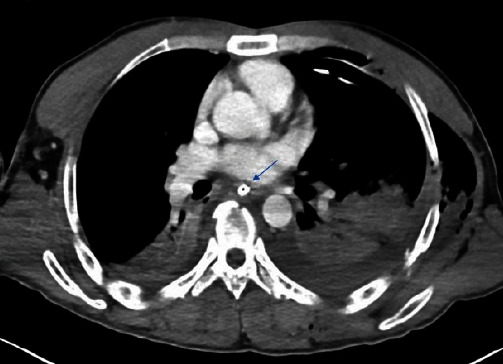
Chest CT scan showing orogastric tube (arrow).

Further investigation revealed a traumatic brain injury (TBI) with brainstem contusion, traumatic mydriasis of the right pupil, a right ear laceration, mandibular fractures, and fracture of the spinous process of C6 and T6 and T7 vertebral bodies. Fractures of left ribs 1–11 and 6–12 on the right side were associated with a left hemopneumothorax. Of note, no mediastinal abnormalities were noted on admission CT imaging (Figure 1).

**Figure 2:**
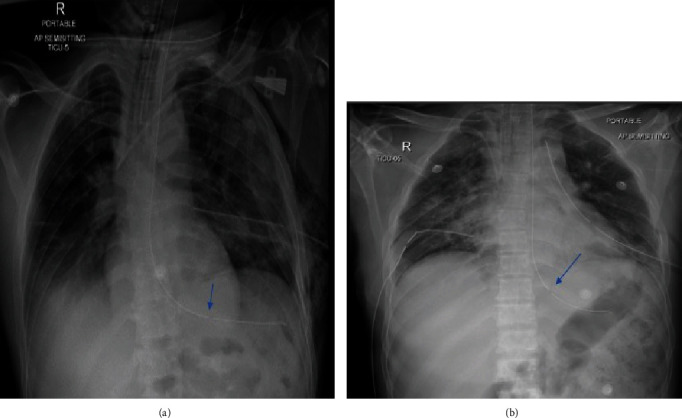
Chest X-ray (a & b) shows endotracheal tube, orogastric tube and left thoracostomy tube: The side hole of the feeding tube seen overlying the gastro-esophageal junction (arrows).

Further management included feeding via a 16 French gauge OGT, the position of which had been confirmed by X-ray (Figure 2), and auscultation as per trauma intensive care unit (ICU) protocol. Later, he was started on Jevity® (Abbott Nutrition) enteral feeding at 80 ml/hour for 24 hours, providing 1,920 kcal/day in accordance with our ICU protocol. Jevity® is a 1 kcal/ml enteral feed with fiber for patients with, or at risk of developing, disease-related malnutrition; it contains a mix of fiber and fructo-oligosaccharides.

A repeat head CT on day 7 revealed a brainstem contusion. A decision was made to insert a percutaneous tracheostomy on the 8th day of admission. Furthermore, 1 liter of transudative pleural effusion was drained via a right-sided 32 French chest drain (inserted using an open technique). The OGT was kept in place during this period. However, the OGT side hole was higher in position (Figure 2).

On day 10, the oral and maxillofacial (OMF) team performed an open reduction and internal fixation (ORIF) of the mandibular para-symphyseal fracture with upper and lower arch bars. During the surgery, the oral endotracheal tube was changed to nasal intubation to facilitate the facial surgery, and the OGT was removed at the same time. Difficultly was encountered by the anesthetist during OGT removal. Video-laryngoscopy revealed whitish solid cheesy material at the upper esophageal orifice, which was manually fragmented and removed using a McGill's forceps. The material was described as “cheesy”, calcified feeding substance formed around the tube and taking the shape of the esophagus. An attempt was made to re-introduce a nasogastric feeding tube (NGT) under direct video-laryngoscopic guidance, but resistance was encountered, and the procedure was abandoned.

On the following day, the patient's oxygen saturation readings dropped to 94–96%, accompanied by tachycardia. A new 16 French NGT was inserted smoothly (one attempt), and the left TT was removed.

**Figure 3:**
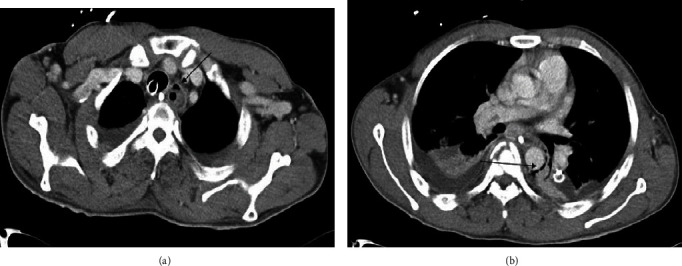
Chest CT scan shows (a) air leak around the esophagus and (b) pneumomediastinum (arrows).

After 2 days, a CT scan pulmonary angiogram was requested to rule out pulmonary embolism due to persistent tachycardia; this revealed pulmonary embolism in the right main pulmonary artery and left upper lobe. Incidentally, a localized area of heterogeneous air and fluid density was noted along the left upper paraoesophageal region with minimal pneumomediastinum, raising the suspicion of an esophageal injury (Figure 3). The gastroenterology team decided to manage the injury conservatively without endoscopy but with antimicrobial cover, as the patient was hemodynamically stable. No organic gastrointestinal pathology was present. The patient made an uneventful recovery from the esophageal injury, without the development of mediastinitis or further complications. However, his functional recovery from the TBI was poor.

## 3. Discussion

Up to the best of our knowledge, in English literature, this is the first published case report of esophageal perforation due to the forceful removal of a wedged OGT secondary to esophageal bezoar formation in a trauma patient.

The risks and benefits of NGT/OGT use should be carefully considered in each patient [3]. Esophageal perforation associated with feeding tube is rare and more often associated with NGT/OGT insertion as opposed to its removal. Resistance during removal of NGTs is not common in practice, even with prolonged use. If it does occur, however, pulling forcefully is not recommended as there may be calcification around the tube (bezoar), resulting from partially regurgitated feeding. Thorough evaluation must be done prior to the removal of any OGT or NGT if resistance is encountered at removal.

Unfortunately, the precipitating factor that could have led to esophageal bezoar formation in our case was the higher position of the NGT (proximal hole at the level of gastroesophageal junction). Such malposition was not detected by the treating physician or radiologist despite routine daily chest X-rays at the time. We hypothesize that this led to the accumulation of enteral feed material in the esophagus which, in the presence of gastric reflux acid, likely caused the casein in the enteral feed to clot as a concretion, so that a bezoar was formed within few days prior to the NGT removal.

Turner et al., showed that Osmolite® (pH of 6.5) was acidified to a pH of less than 5 solidified within 5 minutes [7]. A similar finding was observed when other enteral formulas containing casein were exposed to an acidic environment [6, 8].

Initially, in our case, the OGT insertion was checked routinely by auscultation and by aspiration of gastric contents by suction, and then finally by X-ray. Auscultation, a common practice to check for OGT presence inside the stomach, might falsely reassure if the tube was mistakenly left in the lower part of the esophagus, as the sound bubbling inside the stomach can be transmitted. Radiology remains the gold standard for checking correct NGT placement [9].

Operative exploration of suspected traumatic esophageal perforation is considered gold-standard therapy. Cervical esophageal injuries may be an exception to this rule considering the protection offered to the cervical esophagus by surrounding tissues. Leak of the cervical esophagus may cause less harm than in other segments of the esophagus. Madiba and Muckart, from South Africa, reported a series of 17 patients with traumatic cervical esophageal injury who were managed conservatively; only one patient developed local sepsis [10]. Furthermore, iatrogenic injury in fasting patients makes the passage of organic material through the leak less likely [11].

## 4. Conclusions

Morbidity associated with OGT/NGT insertion or removal is not common and may require a high index of suspicion to be identified. This is especially true if resistance is appreciated while removing the NGT/OGT. Gastroenterology consultation is recommended as early as possible to detect and manage any complications; however, their role was very limited in such stable case. In addition, early CT can be considered for timely recognition of esophageal perforation. Non-operative management may be considered in stable patients, especially if the leak is in the cervical portion of the esophagus. Finally, prevention is better than cure, so being diligence in confirming NGT/OGT position, both radiologically and by measuring the tube length at the nostril/mouth, is the key to avoid its misplacement and complications. This case will raise the awareness of physician for such iatrogenic preventable unfavorable event.

## Data Availability

Data supporting this research article are available from the first author on reasonable request and after a permission from the medical research center at Hamad Medical Corporation.

## References

[1] Yeh J., Saul T., Gingrich A., Wassermann J. (2013). Bezoar. *The Journal of Emergency Medicine*.

[2] Andrus C. H., Ponsky J. L. (1988). Bezoars: classification, pathophysiology, and treatment. *The American Journal of Gastroenterology*.

[3] Yaqub S., Shafique M., Kjæstad E. (2012). A safe treatment option for esophageal bezoars. *International Journal of Surgery Case Reports*.

[4] Myo A., Nichols P., Rosin M., Bryant G. D., Peterson L. M. (1986). An unusual oesophageal obstruction during nasogastric feeding. *British Medical Journal (Clinical Research Edition)*.

[5] Carrougher J. G., Barrilleaux C. N. (1991). Esophageal bezoars: the sucralith. *Critical Care Medicine*.

[6] Marcus E. L., Arnon R., Sheynkman A., Caine Y. G., Lysy J. (2010). Esophageal obstruction due to enteral feed bezoar: a case report and literature review. *World Journal of Gastrointestinal Endoscopy*.

[7] Turner J. S., Fyfe A. R., Kaplan D. K., Wardlaw A. J. (1991). Oesophageal obstruction during nasogastric feeding. *Intensive care medicine*.

[8] Tawfic Q. A., Bhakta P., Date R. R., Sharma P. K. (2012). Esophageal bezoar formation due to solidification of enteral feed administered through a malpositioned nasogastric tube: case report and review of the literature. *Acta Anaesthesiol Taiwan*.

[9] Tho P. C., Mordiffi S., Ang E., Chen H. (2011). Implementation of the evidence review on best practice for confirming the correct placement of nasogastric tube in patients in an acute care hospital. *Int J Evid Based Healthc*.

[10] Madiba T. E., Muckart D. J. (2003). Penetrating injuries to the cervical oesophagus: is routine exploration mandatory?. *Ann R Coll Surg Engl*.

[11] Hauge T., Kleven O. C., Johnson E., Hofstad B., Johannessen H. O. (2019). Outcome after iatrogenic esophageal perforation. *Scand J Gastroenterol*.

